# A Predictive Score Incorporating Clinical, Radiologic, and Hormonal Parameters to Discriminate Lymphocytic Hypophysitis from Non-Functioning Pituitary Macroadenomas

**DOI:** 10.3390/diagnostics15182334

**Published:** 2025-09-15

**Authors:** Ach Taieb, Ines Bouzaouache, Ayoub Gasmi, Aicha Ghachem, Imen Halloul, Wiem Saafi, ElFekih Hamza, Saad Ghada, Yosra Hasni, Houda Mhabrech

**Affiliations:** 1 Faculty of Medicine of Sousse, University of Sousse, Sousse 4002, Tunisia; bzw.ines@gmail.com (I.B.); ayoub.gasmi.007@gmail.com (A.G.); ghachemaycha@gmail.com (A.G.); imen.halloul22@gmail.com (I.H.); wiem.saafi@gmail.com (W.S.); elfekihamza@gmail.com (E.H.); ghada.saad6587@gmail.com (S.G.); y.hasni@gmail.com (Y.H.); mhoudafr@yahoo.fr (H.M.); 2 Department of Endocrinology, University Hospital of Farhat Hached Sousse, Sousse 4031, Tunisia; 3 Laboratory of Exercise Physiology and Pathophysiology, L.R. 19ES09, Sousse 4054, Tunisia; 4 Department of Radiology, University Hospital of Farhat Hached Sousse, Sousse 4031, Tunisia

**Keywords:** hypophysitis, pituitary, macroadenoma, score, diabetes insipidus

## Abstract

**Background/Objectives:** Non-functional pituitary macroadenomas (NFPMA) are uncommon pituitary lesions that do not cause hormonal hypersecretion and are most often discovered at the macroadenoma stage. Consequently, they are more challenging to diagnose, often mimicking other non-secreting sellar masses, among which hypophysitis should be carefully considered. This study aimed to differentiate between non-functioning pituitary macroadenomas (NFPMA) and hypophysitis, two distinct sellar pathologies with overlapping MRI features, by developing a diagnostic score based on clinical, biological, and radiological criteria. **Methods:** We conducted a prospective study, including 56 patients with NFPMA and 16 patients with hypophysitis primarily of the lymphocytic subtype. A total of 31 clinical, biological, and radiological variables were analyzed using univariate and multivariate statistical methods to identify significant predictors and to establish a diagnostic score. **Results:** Nine significant criteria were identified: female sex, headaches, visual disturbances, corticotropic insufficiency, pituitary volume ≤ 7 cm^3^, loss of the posterior pituitary bright spot, cavernous sinus invasion, optic pathway compression, and pituitary stalk thickening. The established score demonstrated significant performance in predicting the diagnosis of hypophysitis (*p* < 0.001; Area Under the Curve = 0.967; 95% CI = 0.926–1). The sensitivity and specificity of this score were 93.8% and 87.5%, respectively, using a threshold ≥0.5. The median score was −2 (interquartile range = [−3.5; 0.5]), with extremes ranging from −6.5 to 9. Among these, pituitary stalk thickening emerged as a key diagnostic indicator. **Conclusions:** This simple and effective multi-parametric score enables rapid and accurate differentiation of hypophysitis from NFPMA, helping to avoid unnecessary surgical interventions and to improve the management of pituitary insufficiencies and may be especially valuable in settings when biopsy is unavailable or risky.

## 1. Introduction

Sellar pathology is highly diverse, with numerous masses encountered in this region [1], which may originate from the sella turcica itself or from parasellar structures, including vascular, meningeal, optic, or bony structures [1,2]. Among these lesions, pituitary adenomas (PA) are the most frequently encountered etiology, accounting for approximately 15% of intracranial tumors and 90% of intrasellar tumors [3]. Functional pituitary adenomas are the most common (75%) and are easily recognizable due to their clinical and biological hypersecretion syndrome. In contrast, non-functional adenomas are less common (35%), do not cause hormonal hypersecretion, and are most often discovered at the macroadenoma stage [4]. Consequently, non-functioning pituitary macroadenomas (NFPMA) are more challenging to diagnose, often mimicking other non-secreting sellar masses, among which hypophysitis should be carefully considered.

Hypophysitis refers to a group of disorders characterized by variable inflammation of the pituitary parenchyma [5]. It is a rare etiology of sellar masses, accounting for less than 1% of pituitary masses and responsible for 0.5% of hypopituitarism cases [6,7,8].

Hypophysitis may involve the anterior pituitary (AH), the posterior pituitary and pituitary stalk (INH), or both simultaneously [5]. It has emerged as a noteworthy cause of intrasellar lesions, with a progressively clearer etiological profile. Hypophysitis represents 0.24% to 0.88% of pituitary disorders, with an estimated annual incidence of 1 case per 9 million inhabitants [8].

No single clinical, biological, or radiological feature can reliably differentiate hypophysitis from NFPMA, given their overlapping clinical and biochemical presentations, as well as their frequent imaging appearance as a solitary sellar mass [9].

The objective of our study was to develop a comprehensive multimodal diagnostic score to guide the differential diagnosis between hypophysitis and NFPMA, thereby optimizing clinical management and reducing the risk of unnecessary or potentially harmful surgical interventions.

## 2. Methods

### 2.1. Study Population and Group Definition

We conducted a prospective descriptive and analytical study on adult patients referred to the Endocrinology department for the evaluation of a sellar mass greater than 10 mm or for another symptomatology that revealed the presence of a sellar mass, in whom the diagnosis of hypophysitis or NFPMA was established.

The study population was divided into two groups based on the confirmed diagnosis:

- Group 1 (G1): NFPMA group. All patients in this group underwent surgical resection of their NFPMA, with histological and immunohistochemical confirmation of its non-secreting nature.
- Group 2 (G2): Hypophysitis group. The diagnosis of hypophysitis was established either histopathologically on surgical specimens from sellar surgery or based on clinical, biological, and radiological criteria suggestive of INH, panhypophysitis, or AH. A presumptive non-surgical diagnosis of hypophysitis was made based on suggestive clinical findings, partial or complete anterior pituitary deficiency, evocative imaging findings, and favorable response to corticosteroid therapy. The simultaneous involvement of both adenohypophysis and neurohypophysis was highly suggestive of an inflammatory etiology.

### 2.2. Sample Size and Patient Inclusion

For G1, we calculated the sample size using the appropriate formula for our study design:

*n* = *z*^2^ · *p* (1 − *p*)/*i*^2^

where

- *n* = sample size;
- *z* = confidence level (1.96 for 95% confidence);
- *p* = prevalence of NFPMA in the general population;
- *i* = margin of error (5%).

Using this formula, we determined a sample size of 25 patients, which we increased to 56 for statistical representativeness. For G2, we included all cases of hypophysitis diagnosed in the Endocrinology department from January 2020 to January 2025, totaling 16 patients.

### 2.3. Selection of Study Criteria

Clinical and radiological criteria were selected based on previously described indicators in the literature for differentiating hypophysitis from NFPMA [10,11]. Additional criteria were identified based on emerging results from our univariate analysis. Criteria selection was predefined to include the most relevant variables for differentiating between these two pathologies. The evaluated criteria were as follows:

#### 2.3.1. Clinical Criteria:

- Demographics: Age (<30 years vs. ≥30 years), sex, pregnancy status (ongoing pregnancy or recent delivery ≤2 weeks postpartum).
- Medical history: Autoimmune diseases, vasculitis, systemic inflammatory diseases, neoplastic or granulomatous conditions.
- Tumor syndrome: Headaches, visual symptoms (decreased visual acuity, bitemporal hemianopsia, homonymous lateral hemianopsia (HLH), ophthalmoplegia, or blindness).
- Signs of anterior pituitary insufficiency, hyperprolactinemia, or central diabetes insipidus (CDI).

#### 2.3.2. Biological Criteria:

- Elevated C-reactive protein (CRP > 10 mg/dL), increased erythrocyte sedimentation rate (ESR > 15 mm/h in men and >20 mm/h in women < 50 years; >20 mm/h in men and >30 mm/h in women ≥ 50 years), leukocytosis (>10,000/mm^3^).
- Anterior pituitary insufficiency: Assessment of corticotropic (Adrenocorticotropic Hormone (ACTH) and 8 AM cortisol; Synacthen test if needed), somatotropic (Insulin-like Growth Factor 1 (IGF-1) and Growth Hormone (GH) stimulation test), gonadotropic (Follicle-Stimulating Hormone (FSH), Luteinizing Hormone (LH), estradiol/testosterone), thyrotropic (Thyroid-Stimulating Hormone (TSH), Free Thyroxine T4 (FT4)), lactotropic (Prolactin (PRL)), and neurohypophyseal function (water deprivation test in suspected CDI).
- Hyperprolactinemia: PRL > 20 ng/mL in men and >40 ng/mL in women; PRL < 200 ng/mL suggests a disconnection hyperprolactinemia.

Hormonal assays were performed using various methods: ImmunoRadioMetric Assay (IRMA) for ACTH, GH, IGF-1, TSH, and PRL; RadioImmunoAssay (RIA) for cortisol, FT4, estradiol, and testosterone; and IRMA or chemiluminescence for FSH and LH.

#### 2.3.3. Imaging Criteria: Magnetic Resonance Imaging:

The following data were analyzed on Magnetic Resonance Imaging (MRI):

- Pituitary mass volume (cm^3^) was calculated using Di Chiro’s formula [12,13] and categorized into two intervals using the 7 cm^3^ threshold, which corresponds to the cut-off value used by Gutenberg et al. [14].
- Signal intensity on T1- and T2-weighted images.
- Enhancement pattern (intense, homogeneous).
- Symmetry of the pituitary mass.
- Absence of the posterior pituitary bright spot.
- Ectopic Antidiuretic Hormone (ADH) storage.
- Pituitary stalk thickening (>4 mm at the optic chiasm/median eminence or >3 mm at the pituitary insertion [15]).
- Cavernous sinus invasion (Knosp grade ≥ 2 [16,17]).
- Compression of the optic pathways.
- Presence of a pseudocapsule
- Presence of a dural tail sign [18,19].
- Sphenoidal sinus mucosal thickening.

### 2.4. Statistical Analysis

Significant criteria from univariate analysis were included in a multivariate regression model to determine independent predictors of hypophysitis. A weighting system was assigned based on odds ratios (OR) and 95% confidence interval (CI). Criteria significantly associated with NFPMA were assigned a negative score (-). Criteria significantly associated with hypophysitis were assigned a positive score (+).

The score’s diagnostic performance was evaluated using a Receiver Operating Characteristic (ROC) curve, with an optimal cutoff, sensitivity, specificity, and Youden index determined.

## 3. Results

We included 56 patients with NFPMA and 16 patients with hypophysitis.

A female predominance was observed in both groups, significantly higher in G2 (*p* = 0.007), with a gender ratio of 0.75 in G1 and 0.06 in G2. The tumor syndrome was significantly more present in G1 (*p* < 0.001), affecting all patients, compared to only 43.75% of G2 patients, mainly consisting of headaches (94.6% in G1 vs. 50% in G2, *p* < 0.001). CDI was significantly more frequent in G2 (62.5% of cases) compared to only 1.78% in G1 (*p* < 0.001) (Table 1).

Corticotropic insufficiency was significantly more frequent in G2 (62.5%) than in G1 (17.8%, *p* = 0.003), and was often observed either as an isolated deficiency or in combination with other hormonal deficits, particularly thyrotropic insufficiency. Conversely, in G1, somatotropic insufficiency was predominant (80.4% vs. 18.8% in G2, *p* < 0.001), frequently presenting as an isolated and asymptomatic impairment, followed by gonadotropic insufficiency (Table 2).

Several radiological criteria were found to be significant in differentiating NFPMA from hypophysitis. A pituitary volume > 7 cm^3^ was significantly more frequent in G1 (42.9% vs. 6.2%, *p* = 0.007). Signal homogeneity before contrast administration and intense, homogeneous enhancement after contrast were more frequent in G2 (*p* < 0.001). Pituitary mass symmetry was exclusively found in G2 (100% vs. 17.9%, *p* < 0.001). Loss of the posterior pituitary bright spot and thickening of the pituitary stalk were more frequent in G2, often associated with CDI (*p* = 0.001 and *p* < 0.001) (Table 3).

Logistic regression identified nine independent predictors distinguishing hypophysitis from NFPMA (Table 4).

**Figure 1:**
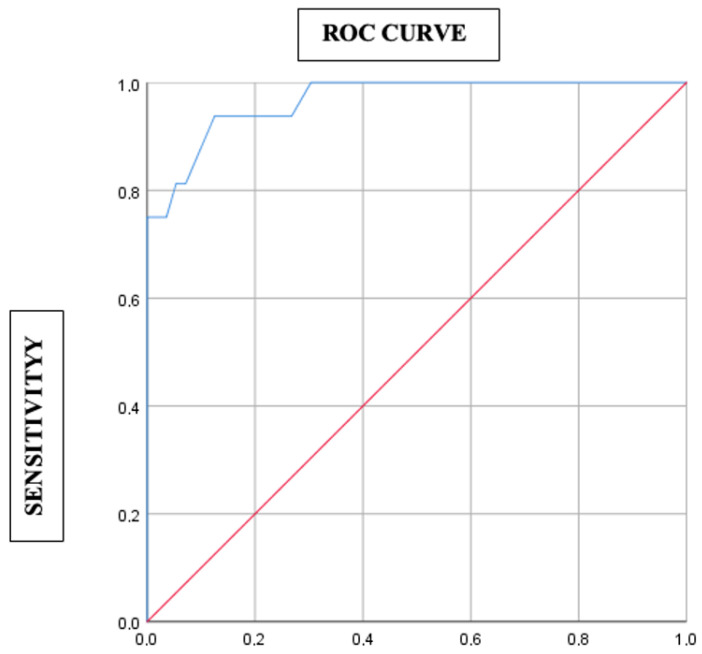
Performance of the established score in predicting the diagnosis of hypophysitis (ROC curve).

The established score demonstrated significant performance in predicting the diagnosis of hypophysitis (*p* < 0.001; Area Under the Curve (AUC) = 0.967; 95% CI = 0.926–1). The sensitivity and specificity of this score were 93.8% and 87.5%, respectively, using a threshold ≥0.5 (Figure 1). The median score was −2 (interquartile range IQR = [−3.5; 0.5]), with extremes ranging from −6.5 to 9 (Table 5).

## 4. Discussion

A total of 31 criteria were analyzed: 9 clinical, 7 biological, and 15 radiological parameters, based on 56 cases of NFPMA (G1) and 16 cases of hypophysitis (G2). The first step involved univariate analysis to identify statistically significant criteria differentiating between the two diagnoses. A subsequent logistic regression analysis determined the independent factors associated with hypophysitis, ultimately leading to the development of a diagnostic predictive score incorporating 9 key elements.

Setting a threshold at 0.5 provided excellent negative predictive value (98%), allowing for the reliable exclusion of hypophysitis in cases of non-secreting sellar masses, thus favoring alternative diagnoses, particularly NFPMA, and facilitating the decision to perform pituitary biopsies when necessary. Conversely, when the score is ≥0.5, the probability of hypophysitis is significant (positive predictive value of 68.2%), warranting further etiological investigations and consideration of first-line medical treatment.

Female sex emerged as a strong predictor of hypophysitis with a weighting of +3 [20]. This criterion was not included in the scores proposed by Gutenberg et al. [14] and Wright et al. [21], despite the observed female predominance in both studies. This discrepancy may be explained by the strong association between female sex and the lymphocytic form of hypophysitis [5,22,23,24], which is likely the most prevalent form in our cohort, whereas other histological subtypes identified in different studies appear to be more gender independent.

Headache was predictive of NFPMA, weighted at −2. This parameter was not included in the scores of Gutenberg et al. [14] or Wright et al. [21]. The absence of a clinical syndrome of hormonal hypersecretion often leads to the diagnosis of NFPMA at an advanced stage, when tumor volume is sufficiently large to cause neuro-ophthalmological symptoms. In contrast, hypophysitis is associated with a smaller pituitary volume and less lateral and suprasellar extension, resulting in a lower incidence of headaches. This suggests that headache mechanisms in hypophysitis may be mediated by biochemical and neuroendocrine processes rather than structural mass effect.

Visual disturbances were also identified as a clinical predictor of NFPMA, weighted at −2. This parameter was not included in the score by Gutenberg et al. [14] but was incorporated into Wright et al.’s score [21]. The frequency and type of visual impairment in hypophysitis appear to be dependent on radiological involvement patterns, with higher prevalence in AH (43%) and panhypophysitis (18%) but lower occurrence in INH (3%) [8]. Visual disturbances are significantly more frequent in NFPMA, typically presenting as bitemporal hemianopsia due to optic chiasm compression. However, acute-onset visual impairment in hypophysitis has been reported and may mimic pituitary apoplexy [23].

Corticotropic insufficiency was the only biological parameter retained in the hypophysitis diagnostic score, weighted at +1. This criterion was absent in both Gutenberg et al.’s [14] and Wright et al.’s [21] scores. Corticotropic axis involvement was the most frequent endocrine dysfunction in our hypophysitis cases and is widely reported in the literature. Some authors [25,26] have proposed that corticotropic cells may serve as the initial antigenic target of the immune response, leading to isolated involvement in the early stages of disease. However, isolated corticotropic insufficiency may also occur in NFPMA, and isolated pituitary hormone deficiencies have been reported in AH [27,28]. The high prevalence of corticotropic insufficiency in hypophysitis may be attributed to its symptomatic presentation, which facilitates early diagnosis.

A small pituitary volume was predictive of hypophysitis, weighted at +0.5. This criterion was absent from Wright et al.’s [21] score but was included in Gutenberg et al.’s [14] score, where a volume > 7 cm^3^ was weighted at +2 as a predictor of NFPMA. NFPMA generally exhibit larger tumor volumes than hypophysitis, a difference that becomes statistically significant at the 7 cm^3^ threshold. This difference likely reflects the indolent progression of NFPMA, leading to a later diagnosis at a stage of hypopituitarism or visual complications. Conversely, hypophysitis is diagnosed earlier due to the rapid onset of hypopituitarism, particularly corticotropic deficiency, which often presents with acute clinical manifestations.

Loss of the posterior pituitary bright spot was predictive of hypophysitis, weighted at +0.5. This criterion was also included in Gutenberg et al.’s [14] score as a feature favoring hypophysitis, weighted at −2. The T1 bright spot of the posterior pituitary remains intact in purely adenohypophyseal involvement but is lost in panhypophysitis and INH [29]. Moreover, the loss of this signal is consistently associated with pituitary stalk thickening, a key radiological feature observed in both panhypophysitis and INH [30].

Cavernous sinus invasion was identified as a predictor of NFPMA, weighted at −0.5. This criterion was also included in Wright et al.’s [21] score, where the absence of cavernous sinus invasion was indicative of hypophysitis and weighted at +2. Cavernous sinus invasion is not always clinically evident [31]. When symptomatic, it most frequently affects the oculomotor nerve (cranial nerve III), followed by the abducens (cranial nerve VI) and trochlear (cranial nerve IV) nerves, leading to ptosis, ophthalmoplegia, and diplopia [32]. Cases of parasellar invasion associated with lymphocytic hypophysitis and presenting as cranial nerve palsies have been described in the literature.

Optic pathway compression on MRI was predictive of NFPMA, weighted at −2. This criterion is the radiological counterpart of the clinical visual disturbances observed. Due to their prolonged course and larger tumor volume, NFPMA more frequently exert mass effects on the optic pathways. The most common extension pattern is suprasellar, exerting pressure on the body of the optic chiasm (formed by the crossing nasal fibers of each optic nerve), resulting in bitemporal hemianopsia. Other sites of optic pathway compression may produce a variety of neuro-ophthalmological presentations.

Pituitary stalk thickening was the most predictive feature of hypophysitis, weighed at +4.5. It was also included in both Gutenberg et al.’s [14] and Wright et al.’s [21] scores, weighed at −5 and +1, respectively, and identified as the strongest predictor of hypophysitis in both studies. In our study, it had the highest weighting (OR: 97.386, *p* = 0.021). Pituitary stalk thickening may occur in isolated INH, typically presenting with CDI and loss of the posterior pituitary bright spot, or in AH or panhypophysitis, often in combination with other pituitary involvement [33,34,35]. It is a highly suggestive feature of inflammatory pathology and is considered the most crucial radiological finding for diagnosing hypophysitis [36]. The anatomical distribution of stalk thickening varies; in AH and panhypophysitis, thickening is diffuse from the optic chiasm to its pituitary insertion, whereas in INH, it is more pronounced at the level of the optic chiasm [30]. Hypophysitis remains the leading cause of pituitary stalk thickening [37] and should be the primary diagnostic consideration, particularly in an appropriate clinical context.

This study has several limitations that should be considered. The most important is the relatively small sample size, which may affect the statistical power and generalizability of our findings. It is important to note that our hypophysitis cohort was predominantly composed of lymphocytic hypophysitis. While key differentiating features like pituitary stalk thickening are common to other subtypes, their demographic and clinical profiles can differ. The performance of this diagnostic score is therefore best validated for distinguishing NFPMA from lymphocytic hypophysitis. Its utility in preoperatively identifying other hypophysitis subtypes should be assessed in future multi-center studies with larger, more histologically diverse cohorts.

## 5. Conclusions

We propose a clinico-radiological scoring system that builds on previously established models while incorporating novel parameters. This scoring system is designed to be easily applicable in routine clinical practice, provided that comprehensive biological assessments are available. Its adoption may facilitate timely diagnostic orientation of non-secreting sellar masses, therefore optimizing their management. By reducing the likelihood of unwarranted surgical interventions, it could contribute to more targeted therapeutic strategies and facilitate the timely treatment of associated pituitary deficiencies.

## Figures and Tables

**Table 1:** Comparison of clinical findings between G1 and G2.

Criterion	G1	G2	*p*
Age (mean ± standard deviation)	50.21 ± 11.97 years	43.5 ± 12.36 years	0.053
Male gender *n* (%)	24 (42.9%)	1 (6.2%)	0.007
Female gender *n* (%)	32 (57.1%)	15 (93.8%)	
Pregnancy *n* (%)	1 (1.7%)	2 (12.5%)	0.122
Autoimmune disease *n* (%)	1 (1.7%)	2 (12.5%)	0.122
Tumoral pathology *n* (%)	2 (3.5%)	0 (0%)	0.7
Granulomatosis *n* (%)	0 (0%)	1 (6.2%)	0.222
Vasculitis *n* (%)	0 (0%)	1 (6.2%)	0.222
Tumoral syndrome *n* (%)	56 (100%)	7 (43.8%)	<0.001
Headaches *n* (%)	53 (94.6%)	8 (50%)	<0.001
Visual disturbances *n* (%)	51 (91.1%)	6 (37.5%)	<0.001
-Bitemporal hemianopia	24 (42.9%)	2 (12.5%)	0.026
-HLH	3 (5.4%)	0 (0%)	1
-Ophtalmoplegia	8 (14.3%)	0 (0%)	0.186
-Blindness	3 (5.4%)	0 (0%)	1
-Decreased visual acuity	22 (39.3%)	4 (25%)	0.294
CDI *n* (%)	1 (1.7%)	10 (62.5%)	<0.001

G1: Group 1, G2: Group 2; HLH: Homonymous Lateral Hemianopia; CDI: Central Diabetes Insipidus.

**Table 2:** Comparison of anterior pituitary hormone assay results in G1 and G2.

Parameter	G1	G2	*p*
**Corticotropic axis**			
Basal cortisol < 50 ng/mL *n* (%)	10 (17.8%)	10 (62.5%)	**0.003**
Peak cortisol < 180 ng/mL *n* (%)	13 (23.1%)	10 (62.5%)	**0.003**
**Thyrotropic axis**			
FT4 < 7 pg/mL *n* (%)	7 (12.5%)	5 (31.3%)	0.32
Mean TSH (μUI/mL) [standard deviation]	2.5 [±1]	1.8 [±1.2]	0.23
**Gonadotropic axis**			
Hypogonadism *n* (%)	8 (14.3%)	0 (0%)	0.122
Mean FSH (mUI/mL) [standard deviation]	9.58 [±9.8]	33.9 [±55.9]	0.222
Mean LH (mUI/mL) [standard deviation]	10 [±11.56]	19.6 [±23.8]	0.323
**Somatotropic axis**			
Mean IGF1 (ng/mL) [minimum–maximum]	97.91 [40–322]	157.83 [8–323.4]	**<0.001**
GH deficiency *n* (%)	45 (80.4%)	3 (18.8%)	**<0.001**
**Lactotropic axis**			
Mean PRL (ng/mL) [standard deviation]	36.5 [±36.4]	23.98 [±25.7]	0.1
Lactotropic insufficiency *n* (%)	0 (0%)	0 (0%)	-

G1: Group 1; G2: Group 2; IGF1: Insulin-like Growth Factor 1; GH: Growth Hormone; FSH: Follicle-Stimulating Hormone; LH: Luteinizing Hormone; FT4: Free Thyroxine; PRL: Prolactin.

**Table 3:** Comparison of MRI findings between G1 and G2.

Radiological Parameter	G1	G2	*p*
Pituitary volume > 7 cm^3^ (%)	42.9	6.2	0.007
Isointense signal on T1-weighted sequences (%)	89.3	100	0.327
Isointense signal on T2-weighted sequences (%)	76.8	93.8	0.169
Homogeneous signal before contrast injection (%)	25	93.7	<0.001
Intense contrast enhancement (%)	1.8	50	<0.001
Homogeneous contrast enhancement (%)	41.1	93.8	<0.001
Symmetry (%)	17.9	100	<0.001
Presence of dural-tail sign (%)	10.7	0	0.327
Pituitary stalk thickening (%)	1.7	62.5	<0.001
Presence of a pituitary pseudo-capsule (%)	16.1	0	0.192
Loss of the posterior pituitary bright spot (%)	25	62.5	<0.001
Ectopic ADH storage (%)	23.2	0	0.059
Invasion of the cavernous sinuses (%)	50	6.3	<0.001
Compression of the optic chiasm (%)	62.5	0	<0.001
Compression of the optic nerve (%)	5.4	0	0.154
Mucosal thickening of the sphenoidal sinus (%)	44.6	0	<0.001

G1: Group 1, G2: Group 2; ADH: Antidiuretic Hormone.

**Table 4:** Multivariate analysis of clinico-radiological and biological criteria for the diagnostic prediction of hypophysitis interpretable in logistic regression.

Criterion	β *	OR	CI_95%_	*p*	Score
**Clinical Criteria**					
Female sex	2.719	15.164	0.255–903.268	0.192	3
Headaches	−2.011	0.134	0.002–8.508	0.342	−2
Visual disturbances	−1.881	0.152	0.005–4.294	0.269	−2
**Biological Criterion**					
Corticotropic insufficiency	1.054	2.869	0.138–59.599	0.496	1
**Radiologiccal Criteria**					
Pituitary volume ≤ 7 cm^3^	0.484	1.623	0.068–38.642	0.765	0.5
Loss of posterior pituitary T1 hyperintensity	0.318	1.375	0.024–78.285	0.877	0.5
Cavernous sinus invasion	−0.436	0.647	0.04–10.557	0.76	−0.5
Pituitary stalk thickening	4.579	97.386	1.975–4801.324	**0.021**	4.5
Optic pathway compression	−1.717	0.18	0.003–9.429	0.396	−2

* β: logistic regression coefficient; OR: odds ratio; CI: confidence interval.

**Table 5:** Diagnostic prediction score of hypophysitis.

Criteria	Points
Female sex	+3
Presence of headaches	−2
Presence of visual disturbances	−2
Corticotropic insufficiency	+1
Pituitary volume < 7 cm^3^	+0.5
Loss of the posterior pituitary bright spot	+0.5
Cavernous sinus invasion	−0.5
Pituitary stalk thickening	+4.5
Optic pathway compression	−2

## Data Availability

The original contributions presented in this study are included in the article. Further inquiries can be directed to the corresponding author.

## Abbreviations

The following abbreviations are used in this manuscript:

ACTH	Adrenocorticotropic Hormone
ADH	Antidiuretic Hormone,
AH	Adenohypophysitis
AUC	Area Under the Curve
CDI	Central Diabetes Insipidus
CI	Confidence Interval
CRP	C-reactive Protein
ESR	Erythrocyte Sedimentation Rate
FSH	Follicle-Stimulating Hormone
FT4	Free Thyroxine T4
G1	Group 1
G2	Group 2
GH	Growth Hormone
HLH	Homonymous Lateral Hemianopsia
IGF1	Insulin-like Growth Factor 1
INH	Infundibulo-neurohypophysitis
IRMA	ImmunoRadioMetric Assay
LH	Luteinizing Hormone
MRI	Magnetic Resonance Imaging
NFPMA	Non-Functioning Pituitary Macroadenomas
OR	Odds Ratio
PA	Pituitary Adenomas
PRL	Prolactin
RIA	RadioImmunoAssay
ROC	Receiver Operating Characteristic
TSH	Thyroid-Stimulating Hormon
